# A Scoping Review on the Management of Open Fractures in African Trauma and Orthopaedics Centres

**DOI:** 10.7759/cureus.68925

**Published:** 2024-09-08

**Authors:** Abdulahi A Zubair, Ridwanullah Abdullateef, Samuel Davis, Adedamola Olaniyi, Inioluwa Joshua, Marvellous Emma-Nwachukwu, Orugbo O Jessie, Ayo-Oladapo Kolawole, Akudo B Umeh, Azeezat A Sunmola, Emmanuel O Oladeji

**Affiliations:** 1 Trauma and Orthopaedics, Surgery Interest Group of Africa, Lagos, NGA; 2 Orthopaedics, Surgery Interest Group of Africa, Lagos, NGA; 3 Urology, Surgery Interest Group of Africa, Lagos, NGA; 4 Surgery, University of Ilorin, Ilorin , NGA

**Keywords:** africa, external fixator, gustilo anderson classification, open fracture, open fracture management, open reduction and internal fixation, open tibia fracture, trauma

## Abstract

An open fracture is when the fractured part of a bone is exposed to the external environment by breaching the overlying soft tissue and skin. Open fractures often arise from high-energy injuries, and the risk of microbial contamination is high. There is a need to understand the management of open fractures in Africa by assessing the overall prevalence of open fractures, the mechanisms of injury, management approaches and outcomes.

A literature search was conducted using PubMed, African Journal Online, and Google Scholar regarding open fracture management in Africa from inception till date. Thirty-nine (39) studies were included in this review. Road traffic accidents represented the majority of all mechanisms of open fractures, with the Tibia being the most affected bone. 320 cases were classified as Gustilo Anderson Type 1, with 487 classified as Type 2. Type 3 was divided into 3A (330), 3B (248), and 3C (34). Most studies recorded the immediate administration of intravenous antibiotics, but tetanus prophylaxis was only given in 13 studies, while initial debridement and washout were done in 35 studies. External fixators and Kirschner wires were most used for initial fixation. Follow-up for patients was between six weeks to 50 months. There were 645 cases of malunion, 83 cases of non-union, and 88 patients who had delayed union. There were 147 cases of wound infection and 119 cases of pin tract infections. Our findings emphasize the need for standardized protocols and robust emergency services to manage open fractures within Africa.

## Introduction and background

An open fracture is when the fractured part of a bone is exposed to the external environment by breaching the overlying soft tissue and skin [1]. Open fractures often arise from high-impact or high-energy injuries and can cause severe, life-threatening injuries. Due to the communication between the fractured part and the external environment, the risk of microbial contamination and foreign body entrapment is high. There is also an increased risk of damage to the surrounding blood vessels, potentially compromising the healing process and increasing the risks of complications such as sepsis, non-union, malunion, etc. [2].

Therefore, the management of such fractures focuses on the prevention and control of infections, adequate union of the fractured ends, and the restoration of function. These targets are achieved through early presentation for treatment, thorough evaluation of the patient and the injury, proper use of antibiotics locally and systemically, adequate wound debridement and closure, and prompt fracture fixation [3]. The management of patients typically involves prehospital review and management, as well as resuscitation upon presentation in a hospital, usually following advanced trauma life support (ATLS) principles. Broadly, a neurovascular assessment of the affected limb, evaluation of the extent of gross wound contamination, wound irrigation and dressing, and assessment for compartment syndrome are undertaken. Patients may require realignment and splint immobilization, administration of intravenous antibiotics and tetanus prophylaxis, and adequate imaging [1,4]. 

In Africa, only a few studies have assessed the management of open fractures holistically, with many only focusing on specific parts of the body. A study from Southern Malawi in 2019 reported an increasing incidence of open tibia fractures, with road traffic accidents being the mechanism of injury in 63% of them. The authors also observed that the infection rate was higher than what was reported in other developing countries, prompting the need to evaluate the overall management outcomes in these countries altogether [5]. In Tanzania, the management of open tibia fractures is affected by low socioeconomic factors and late access to healthcare facilities. These factors lead to delayed hospital admission and initiation of intervention, causing poor management outcomes [6].

A prospective observational study from Nigeria in 2023 reported that 50.5% of the patients presented with Gustilo-Anderson type III open fractures, with high-impact mechanisms, especially motorbike accidents, being the major causes. The authors also reported that the majority of the open fracture cases in their center were handled primarily by a family physician, assisted by medical officers and non-surgical residents. An infection rate of 15% was recorded for type III fractures [7]. A 2024 study in Malawi attempted to improve the management outcomes of open tibia fractures by introducing an intervention guideline for patients' management and outcomes. The authors found that despite the improved knowledge of the clinicians before and after the one-year intervention, there was no significant improvement in patients’ management and outcomes [8].

Africa has various peculiarities concerning open fracture management, but to the best of our knowledge, these peculiarities have not been extensively studied across the continent. This study aims to provide an understanding of the state of open fracture management in Africa by assessing the overall prevalence of the various types of open fractures, their mechanisms of injury, management approaches, and outcomes. Our study evaluates the available data on these across African countries. This will create an opportunity to identify areas that can be improved upon by clinicians and policymakers.

## Review

Methods

The Preferred Reporting Items for Systematic Reviews and Meta-Analyses extension for Scoping Reviews (PRISMA-ScR) checklist was used in this study. A literature search was conducted using PubMed, African Journal Online, and Google Scholar, and it was limited to articles published in English. Articles were included if they were written about the management of open fractures in African trauma and orthopedics centres from inception till date. Articles were selected based on their relevance to the topic. To limit bias, two reviewers working in pairs screened titles and abstracts and subsequently screened full-text studies for inclusion; any lack of consensus was discussed with a third reviewer. A data chart was used to extract relevant data from the included studies. The data included the first author, the title of study, year of publication, country of origin, sample size, the mean age of the population, mechanism of injury, Human Immunodeficiency Virus (HIV) status, affected limb, fracture type, and classification (Gustilo Anderson classification), initial management, initial and definitive method of fixation, outcomes of care (e.g. complications and rates), follow-up period, and others.

Reviews, meta-analyses, abstracts, conference presentations, commentaries, case reports, and letters to editors were excluded. Thirty-nine (39) studies fully satisfied the eligibility criteria and were used in this review. The final search results were exported into Rayyan.ai (a systematic review software, www.rayyan.ai) for the removal of duplicates and the rest of the screening. Zotero™ was used as a reference manager for the referencing of the manuscript. The search strategy was jointly devised by the authors and is summarised below in Table 1.

**Table 1:** Search strategy

Database	Search	Papers
PubMed	(Open fracture africa) (open fracture management Africa)	(331) (183)
Google Scholar	Open fracture management and Africa	The first 100 pages were searched. No additional studies seen.
African Journal Online	Open fracture management and Africa	114 results were seen. No additional study was identified.

Scope of the review

We reviewed all pooled papers and scrutinised them following the inclusion and exclusion criteria as previously agreed by the authors. Duplicates were immediately removed before the screening process, and subsequently, the studies relating to the management of open fractures in Africa were collated for further reviews and screening. Total papers included after duplicates were removed were 841 and papers included after the screening process were 39.

Results

The studies were conducted over 27 years, from 1996 to 2023. In total, 39 studies were included in this review of the management of open fractures in African Trauma and Orthopedic centres. They were from 15 different African countries (Nigeria, Kenya, South Africa, Malawi, Ethiopia, Chad, Cameroon, Tanzania, Ivory Coast, Sierra Leone, Madagascar, Democratic Republic of Congo, Senegal, Uganda, and Niger) with Nigeria contributing the most with 11 studies. In total, 9393 patients were included in this review, with 3059 open fractures across all the studies. The age range of all patients included was 1-90 years. Six (6) of the 39 papers reviewed included 164 HIV-positive patients, while the others did not state the HIV status of patients [9-14].

Mechanism of Injury

**Figure 1:**
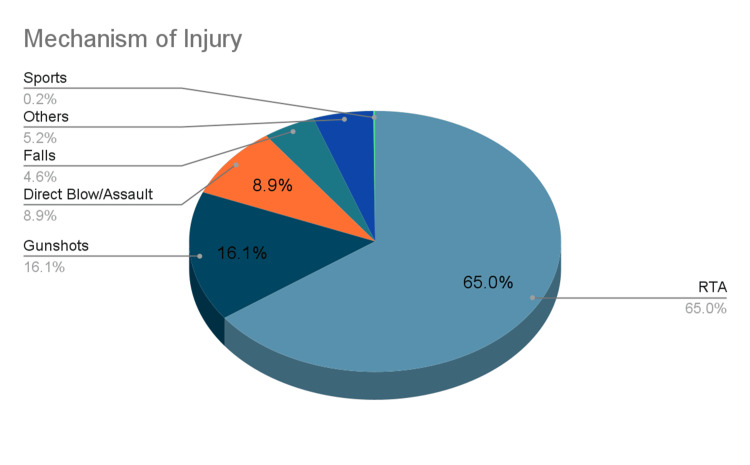
A breakdown of the mechanism of injury and frequency

Across all the studies reviewed, road traffic accidents (also presented as motor vehicle accidents) represented the majority of all mechanisms of open fracture, with 1649 instances of open fracture due to high energy/road traffic accidents. Gunshots, with 409 incidents, came second, while direct blows or assault (225) rounded up the top three. Falls (116), sports (4), and others/unspecified made up 133 cases of open fracture [15]. The mechanism of injury is presented below in Figure 1.

Affected Limb

In all the studies reviewed, the long bone most associated with an open fracture is the Tibia with 1504 cases of open fractures. The femur (347) and Ankle (159) were also frequently fractured. Others are Humerus (55), Radius/Ulna (62), and Others/Undisclosed (312). 

Gustilo-Anderson Classification of Fractures

Three hundred and twenty cases were classified as Gustilo Anderson Type 1, with 487 classified as Type 2. Type 3 was divided into 3A (330), 3B (248), and 3C (34). One hundred and forty-three cases of type Gustilo Anderson Type 3 fractures were seen, but the specific classification (A, B or C) was not given (see Table 3).

**Table 2:** Gustilo-Anderson classification of fractures seen

Gustilo-Anderson Classification	Frequency
Gustilo Anderson Type 1	320
Gustilo Anderson Type 2	487
Gustilo Anderson Type 3A	330
Gustilo Anderson Type 3B	248
Gustilo Anderson Type 3C	34
Gustilo Anderson Type 3 (but unspecified)	143

Two studies used different classification systems: Albright et al. [16] used the Orthopedic Trauma Association and AO Foundation (OTA/AO) classification (240 patients), and Makhubalo et al. [17] used the Muller AO classification (Arbeitsgemeinschaft für Osteosynthesefragen Foundation) (122 patients). The fracture patterns are as follows: Transverse (148), Oblique (163), Spiral (20), Comminuted (321), Segmental (36), Complex and Others (24)

*Initial Management* 

Across all studies reviewed, the following were done as part of initial management following resuscitation. 27 studies recorded the use of intravenous antibiotics in the immediate period of presentation, with 11 studies silent on this [9,11-14,16-37]. In the same vein, tetanus prophylaxis was given in 13 studies, while 25 studies did not specify if this was done. Initial debridement and washout were recorded in all but three studies [17,38,39], with a neurovascular status documented in only six studies [15,17,23,33,34,40]. Plastics review was reported in two cases across all studies reviewed [22,23].

Method of Fixation

In all the included studies, fixation was divided into initial and definitive management. Table 4 provides details about the initial methods of fixation: In 31 studies, external fixators and Kirschner wires (K wires) were used for some patients for initial fixation. In comparison, intramedullary nails (IM nails) were used in 15 studies. In 10 of the studies, primary internal fixation with plates and screws was carried out for patients. 

**Table 3:** Initial method of fixation ORIF: Open reduction internal fixation.

Initial Method Fixation	Frequency
External Fixators and K-Wires	31
IM nail	15
Primary Fixation (ORIF)	10

For definitive surgery, 10 studies managed patients in their cohort with external fixators, Kirschner wires (K wires) and pins, with seven studies [13,16,17,25,30,34,40] employing intramedullary nails as their method of definitive fixation (Table 4). In total, 11 studies managed patients conservatively in the initial phase, while four studies managed some patients non-operatively as a definitive fixation method [11,27,29,38].

**Table 4:** Definitive methods of fixation

Definitive Method Fixation	Frequency
External Fixators, K-wires and Pin	10
IM nail	7

Method of Wound Closure

Ten (10) studies [11,15,18,19,21,22,28,30,33,39] practiced primary closure as part of their management of the cases, while three studies managed patients' wounds with delayed closure. Twenty-one studies (out of 38) did not comment on the method of wound closure.

Other Findings

On the whole, follow-up for patients was between six weeks to 50 months across all studies included in this review. Regarding the outcome of care, there were 696 documented cases of union, 645 cases of malunion, 83 cases of non-union, and 88 patients who had delayed union. There were 147 cases of wound infection and 119 cases of pin tract infections [31]. Osteomyelitis was diagnosed in 33 cases. Two studies included patients who initially presented at a traditional bone setter before secondarily presenting at an orthopedic centre [41,42].

Discussion

The management of open fractures in the African context presents unique challenges, which are highlighted below from our findings in this scoping review.

Mechanism of Injury

Our review identified road traffic accidents (RTAs) as the predominant cause of open fractures in Africa, accounting for 1649 instances. This finding aligns with global trends where high-energy trauma, particularly from RTAs, is a leading cause of such injuries [43,44]. Gunshot wounds were the second most common cause, with 409 incidents, reflecting the socio-political climate in some regions and the prevalence of firearm-related violence [45]. Direct blows or assaults, with 225 cases, underscore the role of interpersonal violence as a significant mechanism. The lower incidence of falls (116), sports-related injuries (4), and other unspecified causes (133) suggest that these mechanisms, while present, are less significant compared to RTAs and gunshots in the African context.

Affected Limb

The tibia was the most commonly fractured long bone, with 1504 cases of open fractures. This high incidence can be attributed to the bone's subcutaneous location, making it more vulnerable to direct trauma [46]. The femur (347 cases) and ankle (159 cases) were also frequently fractured, which may relate to the mechanics of high-energy impacts typically seen in RTAs and falls [47]. The focus on these specific bones is critical for developing targeted management protocols and resource allocation in trauma centres within the region.

Gustilo-Anderson Classification of Fractures

The classification of open fractures using the Gustilo-Anderson system provides insight into these injuries' severity and management complexity [48]. Our review found 320 cases classified as Type 1, 487 as Type 2, and varying numbers of Type 3 fractures (3A: 330, 3B: 248, 3C: 34). Notably, 143 cases of patients recorded as having Gustilo-Anderson type C fractures did not specify the actual type C classification, suggesting potential inconsistencies in reporting and documentation practices. The predominance of Type 2 and Type 3 fractures indicates a high incidence of severe injuries requiring complex management strategies [49].

Management Practices

The review revealed significant variability in managing open fractures across different studies. Intravenous antibiotics were administered immediately in 27 studies, underscoring their critical role in preventing infection [50]. However, the omission of this practice in 11 studies raises concerns about adherence to standard care protocols. Similarly, tetanus prophylaxis was given in only 13 studies, while 25 did not specify its use, indicating potential gaps in preventive care [49]. 

Initial debridement and washout, a cornerstone of open fracture management, were documented in 35 studies, highlighting its recognized importance in reducing infection risk [49]. The documentation of neurovascular status in only six patients points to a critical area for improvement, as thorough assessment is essential for optimal outcomes [51]. The minimal involvement of plastic surgeons suggests a potential underutilization of multidisciplinary approaches necessary for managing complex soft tissue injuries [52]. However, this may be related to the shortage of plastic surgeons in low and middle-income countries [53].

Method of Fixation

Fixation strategies for open fractures varied widely across the studies reviewed. Initial management typically involved external fixators and Kirschner wires (K wires), as documented in 31 studies. These methods are advantageous for providing temporary stability and further assessing and managing soft tissue injuries. Intramedullary nails (IM nails) were employed in 15 studies for initial fixation, reflecting their effectiveness in stabilizing long bone fractures, particularly the tibia [54]. 

For definitive fixation, 10 studies continued using external fixators, Kirschner wires, and pins, while seven studies opted for intramedullary nails. The shift towards IM nails for definitive management underscores their biomechanical advantages and reduced infection risk compared to external fixators [55]. 

Method of Wound Closure

Wound closure techniques were inconsistently reported, with primary closure practised in 10 studies and delayed closure in three studies. According to a study by Moola et al., primary closure, which reduces hospital stay and facilitates early rehabilitation, does not differ in post-operative infection risk or risk of delayed union or nonunion when compared to delayed closure, which allows for thorough debridement but requires prolonged hospital stays and multiple surgeries [56]. The lack of commentary on wound closure in 21 studies suggests a gap in documentation and potentially varying practices that warrant further investigation and standardization.

Infection Rates

Infection rates were significant, with 147 cases of wound infection and 119 cases of pin tract infections. The predominant organisms cultured, *Staphylococcus aureus* and *Clostridium perfringens*, align with common pathogens associated with open fractures [57].

Health Seeking Attitude

Two studies reported cases where patients initially presented to traditional bone setters before seeking formal orthopedic care. This finding reflects cultural practices and the potential delays in receiving appropriate medical treatment, which can adversely affect outcomes [58]. Addressing these cultural factors through community education and improved access to formal medical care is essential for enhancing patient outcomes.

Overall, there is significant variability in the management of open fractures across the studies reviewed which is not the case in the developed countries of the world as they tend to have guidelines to guide clinical practice [59,60]. Table 5 contains all the included studies, their study titles, author list, year of study, and country of study.

**Table 5:** Studies included in the review

Author	Study Title	Year of Study	Country of study
Odatuwa-Omagbemi DO [41]	Open fractures: epidemiological pattern, initial management and challenges in a sub-urban teaching hospital in Nigeria	2019	Nigeria
Olufemi and Adeyeye [18]	Irrigation solutions in open fractures of the lower extremities: evaluation of isotonic saline and distilled water	2017	Nigeria
Ikem et al. [19]	Determinants of management outcome in open tibia fractures in ile-ife	2006	Nigeria
Ayumba et al. [15]	Management of patients with post-traumatic exposed bones at Moi Teaching and Referral Hospital, Eldoret, Kenya	2015	Kenya
Aird et al. [9]	The effect of HIV on early wound healing in open fractures treated with internal and external fixation	2011	South Africa
Bach et al. [10]	Disability can be avoided after open fractures in Africa: results from Malawi	2004	Malawi
Steiner and Kotisso [20]	Open fractures and internal fixation in a major African hospital	1996	Ethiopia
Mathieu et al. [42]	Management of neglected open extremity fractures in low-resource settings: Experience of the French Army Medical Service in Chad	2014	Chad
Fonkoue et al. [21]	Outcome of a 2-stage management of open tibia fracture in a low-income country lacking plastic surgeons: A retrospective cohort study	2023	Cameroon
Veldman et al. [11]	The importance of anatomical reduction in the functional outcome of open ankle fractures	2020	South Africa
Omoke et al. [22]	Analysis of risk factors for wound infection after extremity fracture caused by machete cut in a resource-limited setting	2022	Nigeria
Wisniewski and Radziejowski [23]	Gunshot fractures of the humeral shaft treated with external fixation	1996	South Africa
Clelland et al. [24]	The epidemiology and management of tibia and fibula fractures at Kilimanjaro Christian Medical Centre (KCMC) in Northern Tanzania	2016	Tanzania
Albright et al. [16]	Delays to surgery and coronal malalignment are associated with reoperation after open tibia fractures in Tanzania	2020	Tanzania
Salisu et al. [25]	Management of Gustilo and Anderson type i and ii open tibial fracture using delayed primary nailing: An assessment of clinical and radiological outcome	2021	Nigeria
Semenya et al. [26]	An Evaluation of the Use of External Fixator in the Management of Open Tibial Shaft Fractures at a Tertiary Hospital in Pretoria, South Africa	2023	South Africa
Nieuwoudt et al. [12]	Short-term results of grade III open tibia fractures treated with circular fixators	2016	South Africa
Ikem et al. [19]	Open fractures of the lower limb in Nigeria	2001	Nigeria
Kouassi et al. [28]	Locally developed external fixators as definitive treatment of open tibia diaphyseal fractures: a clinical prospective study conducted in Ivory Coast	2021	Ivory Coast
Makhubalo et al. [17]	Early outcomes of surgically managed civilian gunshot femur fractures at a level one trauma unit in Cape Town, South Africa: a retrospective review	2022	South Africa
Lawal et al. [38]	Monolateral frame external fixators in the definitive management of open limb fractures in North-western Nigeria	2016	Nigeria
Sitati et al. [29]	Early bacterial cultures from open fractures-differences before and after debridement	2017	Kenya
Enweluzo et al. [61]	Morbidity of open tibia fractures in Lagos, Nigeria	2015	Nigeria
Kalande FM [30]	Treatment outcomes of open femoral fractures at a county Hospital in Nakuru, Kenya	2018	Kenya
Ogundele et al. [62]	Results of operative fixation of fractures of the ankle at a tertiary hospital in a developing country	2013	Nigeria
Wariboko et al. [31]	The Trend of Wound Microbial Characteristics in Open Fractures at the University College Hospital, Ibadan	2017	Nigeria
Graham et al. [13]	Fracture healing in patients with human immunodeficiency virus in South Africa	2021	South Africa
Harrison et al. [14]	Open fractures of the tibia in HIV positive patients: a prospective controlled single-blind study	2004	Malawi
Perdomo-Lizarraga et al. [32]	Usefulness of external fixation and reverse Sural fasciocutaneous flap: Treatment of grade III B open tibial fractures in resource-limited settings	2024	Sierra Leone
Ramampisendrahova et al. [33]	Management of Open Fractures in Low-Income Countries: a Daunting Task	2021	Madagascar
Seyni and Mohamed [34]	Treatment of open leg fractures by intramedullary nailing	2016	Niger
Olasinde et al. [40]	Outcomes of the treatment of gunshot fractures of lower extremities with interlocking nails	2012	Nigeria and the West Indies
Nana et al. [35]	Epidemiological and Clinical Pattern of Open Fractures of Long Bones of the Lower Limbs in the South-West Region of Cameroon: A 5-Year Review	2021	Cameroon
Sidibe et al. [36]	Open Fractures of Limbs by the Bite of Domestic Donkeys: An Unusual Aetiology	2022	Senegal
Alidou et al. [37]	Intramedullary nailing of type I and type II open leg fractures after 6 hours at Yopougon Teaching Hospital	2016	Ivory Coast
Toha et al. [63]	Exclusive Fibula Osteosynthesis for Treating Open Fractures Gustillo I-III B of the Distal Half of the Leg Bones in a Resources-Limited Setting	2023	Democratic Republic of the Congo
Ekure et al. [64]	Short-Term Outcomes of Treatment of Open Fracture of Long Bone Using Surgical Implant Generation Network Nail at Kumi Orthopaedic Center	2020	Uganda
Mathieu et al. [42]	Management of Gustilo type IIIB open tibial shaft fractures with limited resources: experience from an African trauma center	2021	Senegal
Dessie M [39]	Childress's Technique of Calcaneo-talotibial Steinmann Pin Fixation in Unstable Open Fracture Dislocation of the Ankle Joint at Dilchra Referral Hospital in Ethiopia	2012	Ethiopia

## Conclusions

The findings from this scoping review emphasize the need for standardized protocols and improved documentation in the management of open fractures within the African region. The predominance of high-energy trauma, particularly from RTAs and gunshots necessitates the need for robust emergency response systems and widespread trauma care infrastructure. The variability in management practices, particularly regarding antibiotic use, tetanus prophylaxis, and neurovascular assessment, highlights areas for clinical improvement and training as well as national or continentally agreed guidelines for the management of open fractures. The role of the government in achieving the above cannot be overemphasized. Future research should focus on establishing best practice guidelines and enhancing multidisciplinary collaboration to improve outcomes for patients with open fractures in Africa.

Despite the comprehensive nature of this study, we had some limitations. First, we included articles only in the English language and may have missed some papers from non-anglophone countries. Additionally, we searched three databases, we may have missed potentially relevant papers from others. Finally, the non-disclosure of some papers on specifics of management, and a lack of a specific standardized method of measuring outcome or reporting practices means that there are limitations to the outcome analysis.
